# A Computational Study of Stimulus Driven Epileptic Seizure Abatement

**DOI:** 10.1371/journal.pone.0114316

**Published:** 2014-12-22

**Authors:** Peter Neal Taylor, Yujiang Wang, Marc Goodfellow, Justin Dauwels, Friederike Moeller, Ulrich Stephani, Gerold Baier

**Affiliations:** 1 School of Computing Science, Newcastle University, Newcastle upon Tyne, United Kingdom; 2 College of Engineering, University of Exeter, Exeter, United Kingdom; 3 School of Electrical & Electronic Engineering, Nanyang Technological University, Singapore, Singapore; 4 Department of Neuropediatrics, University Medical Center Schleswig-Holstein, Kiel, Germany; 5 Cell and Developmental Biology, University College London, London, United Kingdom; University of California, Riverside, United States of America

## Abstract

Active brain stimulation to abate epileptic seizures has shown mixed success. In spike-wave (SW) seizures, where the seizure and background state were proposed to coexist, single-pulse stimulations have been suggested to be able to terminate the seizure prematurely. However, several factors can impact success in such a bistable setting. The factors contributing to this have not been fully investigated on a theoretical and mechanistic basis. Our aim is to elucidate mechanisms that influence the success of single-pulse stimulation in noise-induced SW seizures. In this work, we study a neural population model of SW seizures that allows the reconstruction of the basin of attraction of the background activity as a four dimensional geometric object. For the deterministic (noise-free) case, we show how the success of response to stimuli depends on the amplitude and phase of the SW cycle, in addition to the direction of the stimulus in state space. In the case of spontaneous noise-induced seizures, the basin becomes probabilistic introducing some degree of uncertainty to the stimulation outcome while maintaining qualitative features of the noise-free case. Additionally, due to the different time scales involved in SW generation, there is substantial variation between SW cycles, implying that there may not be a fixed set of optimal stimulation parameters for SW seizures. In contrast, the model suggests an adaptive approach to find optimal stimulation parameters patient-specifically, based on real-time estimation of the position in state space. We discuss how the modelling work can be exploited to rationally design a successful stimulation protocol for the abatement of SW seizures using real-time SW detection.

## Introduction

Epilepsy is a chronic neurological disorder characterised by recurrent seizures. In children, several types of seizures display generalised rhythmic spike-wave (SW) discharges in the electroencephalogram (EEG). Spike-wave seizures may appear benign, as in the case of typical childhood absence. However, these may occur frequently, and patients show increased co-occurrence of behavioural, cognitive, and linguistic disorders [1], [2]. Anti-epileptic drug treatment is available but due to the chronic nature of the disorder patients often suffer from side-effects that impact their quality of life [3]. Spike-wave seizures frequently lack pathological neuroradiological abnormalities and invasive treatments such as surgical intervention are typically not indicated. Alternative means to reduce seizure activity are therefore sought.

**Figure 1:**
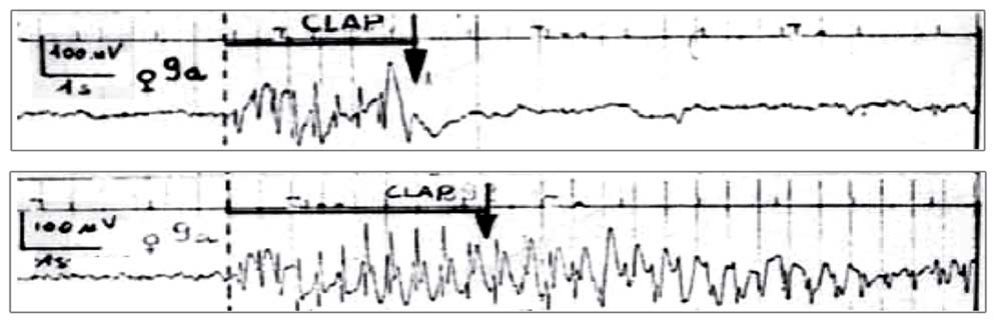
Successful and unsuccessful auditory stimulation. Clinical EEG recordings of successful (upper panel) and unsuccessful (bottom panel) seizure abatement by an auditory stimulus (arrow). Figure modified from [8] with permissions.

The control or suppression of epileptic seizures using stimulus perturbations offers a potential alternative to anti-epileptic drugs. Investigations of the potential of brain stimulation to abort seizures have been undertaken in both humans and animal models of epilepsy [4] including epilepsy associated with SW seizures. In animal models of generalised spike-wave seizures, electric, magnetic and auditory stimuli have been shown to abate seizures [5], [6], whereas electrical vagus-nerve stimulation was less successful [7]. In humans, brief auditory stimuli at SWD onset led to a reduction of average seizure length in about 57% of cases in a study with 19 patients, but a significant number of failed stimulations were also reported [8]. Fig. 1 shows examples of a successful and an unsuccessful application of an auditory stimulus during a SW seizure from this study [8]. In a single-case report, transcranial magnetic stimulation repeated at 5 Hz reduced seizure duration in a pediatric patient [9]. These variable results indicate that stimulation protocols for spike-wave seizures may not yet be optimal. In addition, the reasons why the success of stimulation varies greatly are not well understood [6].

The design of effective and efficient stimulation protocols requires a rational approach, incorporating knowledge of the mechanisms underlying the generation of seizures and their electrographic signatures. Apart from animal experimental studies, mathematical models are an ideal means to explore these mechanisms and to test the potential effects of different perturbations before their application in patients (see e.g. [10]). A number of mathematical models were developed to describe the abnormal SW rhythm [11]–[15]. The model of Breakspear et al. [12] (which builds on [11]) explicitly accounts for the thalamocortical interactions which are crucial for the generation of SW seizures in rodent models [16], [17] and in humans (e.g. see [18]–[20]). However none of these models have been used to investigate stimulation in SWD.

Lopes da Silva et al. suggested to view epileptic seizures from the perspective of dynamical diseases [21]. They argued that while the focal onset of so-called partial seizures is consistent with the slow modulation of a systems parameter, the dynamic mechanism for generalised seizures might be different. They suggest that SW seizures occur from a normal background dynamics in a bistable setting. I.e. the seizure state coexists with the background state and the transition to the seizure state is induced by the presence of noisy fluctuations, e.g. from subcortical input to the cortex. This hypothesis was supported by a mechanistic computational model of the thalamo-cortical loop dynamics [22] and the SWD seizure duration statistics obtained from rat models and humans [23].

Assuming bistability, the success of the single-pulse stimulation can be explained by the presence of the so-called basin of attraction of the background state. This basin is the structure in state space that is associated with normal background dynamics. Whenever the system (in our case the brain electric activity) assumes the background state, it will be contained within the basin structure. During the seizure, the system assumes a trajectory outside of this basin. In these terms, the goal of stimulation is therefore to apply a single pulse perturbation such that the brain activity returns to a state within the basin of attraction of its background state.

For the case of a coexistence of the seizure and the background state it was proposed that brief single-pulse stimulation might be sufficient to abort the seizure [24]. This was numerically confirmed for the thalamo-cortical model of coexisting background and seizure states [22]. The authors showed that an appropriate single pulse stimulus can abate an abnormal oscillatory state. That study found optimal sets of stimulation phases and amplitudes in noise-free, deterministic simulations (see Fig. 6c of [22]). However, previous models studying single pulse stimulation in bistable systems either did not incorporate the complexities of the SW waveform morphology, or study the impact of noise on the resulting stimulation. Furthermore, a detailed analyses of the state space (basin of attraction) have not been shown in previous models, often due to their high dimensionality.

In the following we investigate the basin of attraction of the background state in a minimal thalamo-cortical model of SW seizures. We use the model to examine the effect of single pulse stimulation in the absence and in the presence of noise. From the results we derive some suggestions for the practical application of stimuli during absence seizures based on real-time detection of SW.

## Results

### Spontaneous spike-wave dynamics

**Figure 2:**
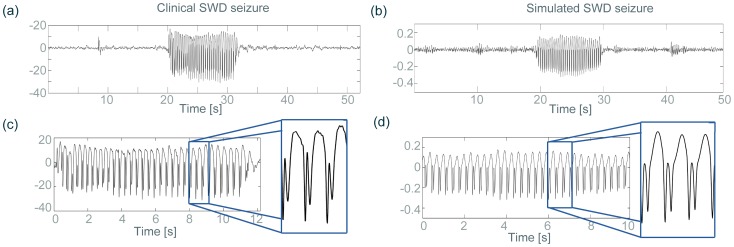
Comparison between clinical and simulated EEG. The clinical (left) and simulated (right) EEG are compared in various properties, such as the long-term time series (a,b), and seizure waveforms (c,d).

Fig. 2 (a) shows a clinical recording of a typical SWD seizure from a single EEG electrode. There is an apparently spontaneous transition from a normal irregular background state to an abnormal seizure state with large amplitude regular oscillations. The seizure stops abruptly after about 11 seconds and is followed by continued normal background activity. To account for this paroxysmal dynamics, we use the minimal model (Equation 4) of thalamo-cortical interactions. The model describes the temporal evolution of the state of four variables corresponding to the activity of populations of (i) cortical pyramidal neurons (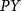), (ii) cortical inhibitory interneurons (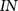), (iii) thalamo-cortical neurons (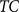), and (iv) inhibitory (thalamic) reticular neurons (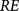) [25] (see section Model and Methods for details). The model can account for the background state of normal activity and the rhythmic SW state of abnormal activity. Parameters are set such that the background state coexists with the SW state in the absence of noisy input. The addition of noise (simulating e.g. irregular subcortical input to the cortex) results in irregular background activity and occasional noise-induced transitions to large-amplitude SW rhythms. Fig. 2 (b) shows a simulated time series for comparison with the clinical recording Fig. 2(a) . In this setting the simulated paroxysms have durations between 10–15 seconds which is common for clinical absence seizures in humans [23]. Fig. 2(c) shows a zoom into the EEG seizure state and the morphology of the SW waveform with a duration of approximately 300 msec. A zoom into the simulated seizure dynamics (Fig. 2 (d)) reveals qualitative similarity of the SW complex, its large amplitude and a duration of about 300 msec. The model thus correctly reproduces the proposed mechanism of a dynamical setting where the background state and the seizure state coexist, and are in close vicinity to each other such that noisy input induces sudden transitions to the seizure state and back again [21].

### The basin of attraction

In the deterministic bistable model (Eq. 1), two attractors coexist (background and SW). Hence the state space can be separated into two distinct areas, each associated with an attractor. The set of all possible states evolving towards the background state is termed the basin of attraction of the background. Similarly, the SW basin of attraction is the set of all states that evolve towards to SW attractor. These two sets of states are distinct in state space and separated by a manifold, termed a separatrix. Unlike all previous thalamo-cortical models of SW, the current model with its four variables allows the comprehensive study of the basin of attraction of the background state (which is also four dimensional). In the background state, small perturbations (within the background basin of attraction) will not lead to a qualitative change of dynamics. Stronger perturbations (beyond the separatrix) result in a transition to the rhythmic spike-wave seizure state. Analogously, when on the SW attractor, a perturbation beyond the separatrix is required for a return to the background state. Thus the basin of attraction of the background should be the target for seizure abating stimuli. In the following, we are therefore interested in the basin of attraction of the background.

**Figure 3:**
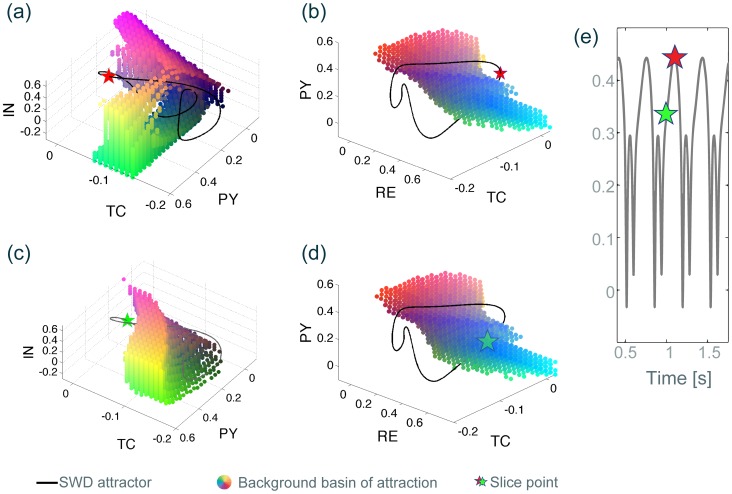
Three dimensional slice through the four dimensional basin of attraction of the background state. Black line indicates the SWD attractor. Stars show the slice point positions. Coloured dots are located in the basin of attraction of the background state in three dimensional state space. Colouring is only included to enable better three dimensional visibility of the geometry of the basin. Red, green and blue intensities encode the three principal axes of the 3D plot. Additive colouring is used to plot off-axis positions (e.g. red & green contribute to the yellow areas between the first and second axis). The SWD basin of attraction is not specifically shown, as it is simply the part in state space that is not the background basin. **(a)** and **(c)** are three dimensional 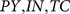 slices through the four dimensional state space. The slice point in the fourth dimension corresponds a single value of 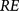 on the SWD attractor at time 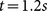 (red star) for (a) and 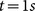 (green star) for (c). **(b)** and **(d)** 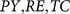 slices with the slice point corresponding to a single value of 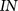 on the SWD attractor at time 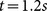 (red star) for (b) and 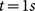 (green star) for (d). **(e)** Corresponding time series showing the slice points. 3D Matlab.fig files are available for (a) and (b) in S1 File & S2 File.

We have numerically determined this basin of attraction for our model in the absence of noise by systematically scanning points in the four dimensional state space of the model. Points that evolve towards the background state are points in the basin of attraction of the background. The ensemble of all detected points in the basin gives an impression of the geometry of the basin. While the four dimensional geometry cannot be visualised fully in a single image we can study different projections to lower dimensions. Essentially, we can take three dimensional slices through the four dimensional basin structure by keeping one model variable constant. In such a three dimensional projection, all the points that evolve towards the background state can be shown in a 3D plot, and they all share the same initial condition value in one variable. Figs. 3 (a) and (b) show two projections of the basin points in (pseudo-) 3D plots. The actual matlab 3D plots are shown in S1 File & S2 File. The projection of the basin points in the pyramidal-inhibitory-thalamocortical population space (PY-IN-TC, keeping RE constant) in Fig. 3 (a) appears to embrace the spike-wave attractor and is indeed located in the vicinity of it. The projection in the pyramidal-thalamocortical-reticular population space (PY-TC-RE, keeping IN constant) in Fig. 3 (b) shows how the spike-wave attractor surrounds the basin, in agreement with the schematic picture in [21]. However, these are solely projections for a single value in the fourth dimension. The projection points in the fourth dimension (i.e. the value of the reticular population when projecting into the PY-IN-TC space, and the value of the inhibitory population when projecting into the PY-TC-RE space) in these figures were chosen to be points on the SW attractor, since we ultimately wish to perturb into the background basin from the SW attractor. Such a projection is essentially showing the basin of the background viewed from this point on the SW attractor. In other words, when on a particular point of the SW attractor, we show the basin of attraction of the background (fixed point) in two projections. Fig. 3 (c) and (d) additionally show the basin from a different projection point on the SW attractor. The shape and size of the basin in this projection point has changed considerably. Fig. 3 (e) shows these two projection points in a time series of SW oscillations (indicated by the two stars).

A more complete view of the basin can be obtained if the same projection is plotted during the evolution of the system on the spike-wave cycle, i.e. for a range of values of the fourth variable. This time-dependent projection can be seen in the S1 Movie. The video demonstrates how the three dimensional projection of the basin changes both size and form during the SW cycle. Specifically, there is a larger volume of the basin in the projection during the slow wave component as compared to the time while the system performs the spike.

Upon the inclusion of noise, the model exhibits spontaneous episodes of seizure arising autonomously from the background, comparable to the clinical occurrence of SW seizures (see Fig. 2). In the presence of noise input, the basin of attraction of the background fixed point becomes less well defined compared to the deterministic case (Fig. 3). Particularly near the basin boundary trajectories from a particular point in state space can develop differently under different noise-inputs. Hence, it is difficult to unambiguously decide whether that particular state space point leads to trajectories returning to the background fixed point or not. In this case, the either/or decision of the deterministic case is replaced by a likelihood. This likelihood of a particular point in state space to return to the background dynamics can be approximated by simulating repeated trials from the same initial conditions with different noise inputs. This “return probability” is 0 for points that never return, 1 for points that always return and a value between 0 and 1 for points are found to lead to both the background and the seizure state.

**Figure 4:**
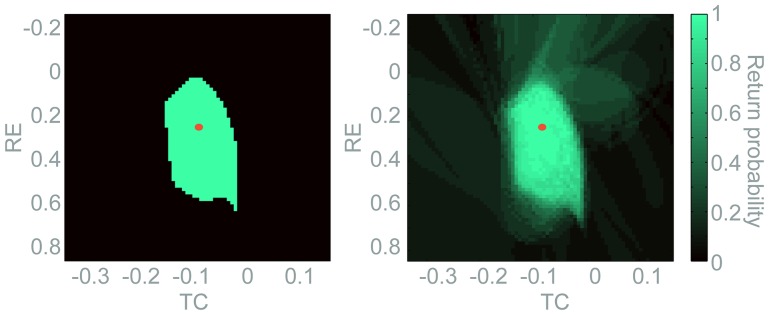
Return probability in a 2D slice through state space. Two dimensions of the four dimensional state space is visualised in the deterministic (left panel) and noise-driven (right panel) models. 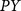 and 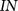 are fixed at the value of the background fixed point. Return probabilities (colour code) are scanned in the 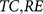 variables. The red dot marks the position of the background fixed point.

In Fig. 4 we compare projections of the return probability in a two dimensional state space in the deterministic case (left) and under noise input (right). The two dimensional 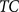-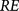 state space is taken at the 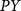 and 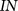 values of the fixed point. In the deterministic case (Fig. 4, left), there is a well-defined boundary between the basin of attraction of the background (green) and the basin of SW dynamics (black). It was shown that clinical SW dynamics is dominated by deterministic dynamics [26] and it is therefore likely that the basin boundary will preserve major features of the deterministic case in the presence of noise. Simulating the model with noise input we find that the structure of the basin of attraction from the deterministic case is indeed still discernible (Fig. 4, right). However, the boundary is now fuzzy and a gradient in return probabilities can be observed. The degree of “fuzziness” depends on the noise amplitude and the dynamics of the system. Importantly, however, the core of the deterministic basin retains a high return probability.

### Stimulation of deterministic spike-waves

In the deterministic case the model set in the background state does not show spontaneous paroxysms. Similarly, when starting the deterministic model on the bistable SW attractor, it does not return to the background. To study the effect of stimulation we therefore start the model in the SW rhythm and apply single-pulse stimuli.

In a clinical setting it is unlikely that individual neural populations in the cortex or thalamus can be addressed individually. We therefore assume that a simulated stimulus affects the activity of both cortical neural populations (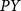 and 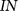) simultaneously (e.g. modelling a TMS pulse). In essence, we thereby fix the direction of the stimulus in state space and allow its timing and amplitude to vary. We will demonstrate later that fixing the direction does not restrict the generality of the investigation.

**Figure 5:**
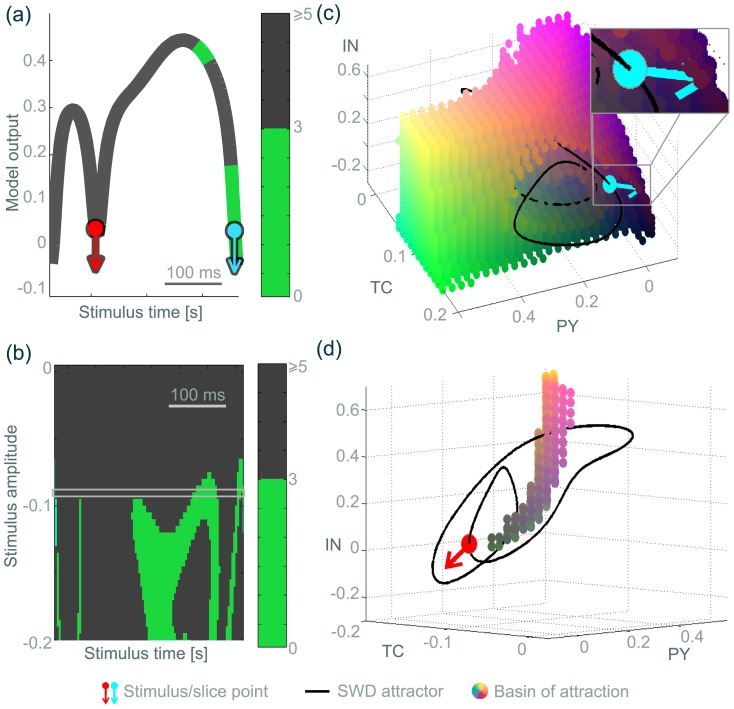
Single pulse stimulation in a deterministic SWD system. **(a)** Colour coded time series of one cycle of SWD. The green colour indicates a return to the background fixed point if stimulated at the colour-coded position (using a fixed stimulus amplitude). Blue/red arrows indicate stimulation points in (c)/(d). **(b)** Colour coded map of stimulation amplitude and timing in the same SWD cycle. The same colour code as in (a) has been used. The particular amplitude used for (a) has been outlined in a grey box. **(c)** Basin of attraction of the background state (coloured dots) in the 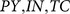 projection for the stimulation point on the SWD cycle is shown together with the SWD attractor (black line). Blue arrow indicates the successful stimulus at this point, as it points into the basin of attraction. **(d)** Same as (c). Red arrow indicated the unsuccessful stimulus at this point as it does not point into the basin. Notice the change in axes between (c) and (d), the figures are rotated to aid visualisation, however, the stimulus direction is the same. 3D Matlab.fig files are available for (c) and (d) in S3 File & S4 File.

Fig. 5 (a) shows a time series for one deterministic SWD cycle where a stimulus of fixed amplitude was applied at different phases of the cycle. I.e. we took different time points in a SW cycle and applied the same stimulus at those time points. Stimulation outcome is labelled as successful and coloured in green when the system's behaviour goes to background activity. If the stimulus is unsuccessful, the colour code is dark grey (SW continues). Hence the result is a colour coding allocated for each time point of stimulation. In turn, these time points can be mapped to the SW time series, and hence the resulting figure is a colour-coded time series. There are two phases during the slow wave component where the stimulus to the cortical populations stops the SW oscillations. For the majority of the time series (shown in dark grey), this particular single pulse stimulus does not stop the model seizure.

**Figure 6:**
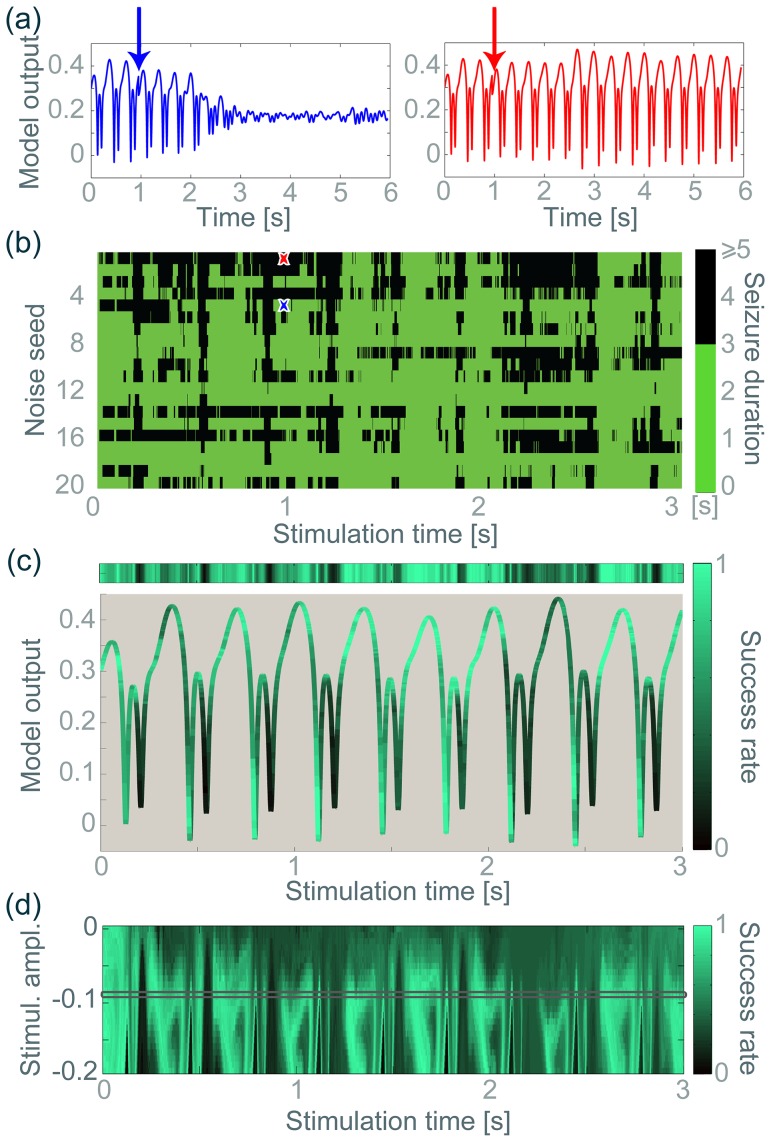
Stimulation in the noise-driven system. **(a)** Two examples of the same simulation timing and amplitude in the same seizure, but with varied outcomes. Different noise inputs were used after the stimulation for the two examples. **(b)** The effect of different noise inputs (y axis) is scanned depending on the ensuing seizure duration (colour code). As in (a), different noise inputs were used following the stimulation. The stimulus amplitude was constant (−0.0825) for the whole scan. **(c)** The top bar indicates the success rate (derived from the data in (b)) at each stimulation time point, which is then applied as a colour code to the actual SWD time series. **(d)** The success rate is also scanned for different stimulation amplitudes. In essence the strip in (c) is a row in (d), indicated by a grey bar.

Fig. 5 (c) shows a state space representation of an exemplary successful stimulation (marked at a blue arrow in Fig. 5 (a)). The basin of attraction of the background state is shown together with the SW cycle (thick black line). The blue arrow indicates the stimulation direction and amplitude. This stimulation indeed targets the basin of attraction, as shown in the zoom in the upper right corner of (c), and thereby leads to SW termination. In contrast, Fig. 5 (d) shows an exemplary unsuccessful stimulus, indicated by the red arrow (corresponding to the red arrow in the time series view in (a)). This time the stimulation misses the basin of attraction, and the SW activity continues despite of the stimulation. The apparent change of the basin of attraction from (c) to (d) is, as explained in the previous section, due to the three dimensional projection. The actual basin of attraction is a fixed four dimensional structure and does not change.

In Fig. 5 (b), additional to scanning the stimulation time points, we also varied the stimulation amplitude. The scan result (success of stimulus) is plotted depending on the two scanned parameters, stimulation time and stimulus amplitude. The color coding is as in (a): the green denotes stimulation parameter combinations for which stimulation terminates SW dynamics, and dark grey indicated unsuccessful stimulation parameters. There is a minimum stimulation amplitude below which no SW termination is seen (around 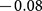) and for stronger stimuli there can be up to six phases on a single cycle during which termination is achieved. Only one of these phases is broad and could be considered as a candidate for an optimal stimulation protocol. Note, that the width of the area changes with stimulation amplitude.

The grey box indicates the stimulation strength applied to obtain the colour coding in Fig. 5 (a), which was mapped to the time series. Here we observe that stimulation during the first green phase in Fig. 5 (a) with both a weaker and a stronger stimulus may be unsuccessful. To reiterate, it is the geometry of the basin of attraction that determines the outcome of a stimulation. This underlines that stronger stimulation does not necessarily lead to better success. On the contrary, we found cases, where a stronger stimulus not only did not abort the seizure but substantially prolonged it compared to the unstimulated case.

In summary, successful SW abatement in the deterministic case by a single pulse stimulus critically depends on the phase/timing and the amplitude of the applied stimulus. In the case of realistic spike-wave morphology the situation is more complex than previously found in a model where the seizure waveform did not have spike-wave morphology but was a simple oscillation [22]. The fixed stimulus direction does not change our result in this section qualitatively. Using different stimulus directions, we still find phases of successful stimulation and unsuccessful stimulation depending on the stimulus amplitude. The success still depends on if the stimulus reaches the basin of attraction of the background state. Only the colour code positions in Fig. 5 (a) and the map in Fig. 5 (b) would have a different structure (data not shown).

### Stimulation in the noise-driven model

To investigate the impact of stimulation in the noise-driven setting, we systematically scan the stimulation timing and amplitude as in the previous section. However, as explained, unlike in the deterministic case, where only the target state space position determines the stimulation outcome, the added noise is now an additional factor influencing the stimulation outcome. In fact, we know that even on the SW trajectory, the probability of returning to the background is non-zero. Hence simulated seizures terminate eventually under noise-input, even without stimulus. To account for this effect, we vary the noise input after the stimulus in repeated simulations and measure the probability of reaching the background state with a given stimulus, whereby we approximate the return probability of the stimulus target position.

Fig. 6 illustrates the variation of stimulation responses depending on the noise input. Fig. 6 (a) shows two exemplary time series following the same stimulus during the same seizure, but using two different noise inputs after the stimulus. In the left hand panel the seizure is successfully terminated, whilst in the right hand panel the SW continues. Compare this to the outcome of the clinical situation displayed in Fig. 1.

To investigate the effect of the noise-input on stimulus success systematically, we scan the stimulus timing (for the same stimulus amplitude) over repeated trials using different noise-inputs after the stimulus. Fig. 6 (b) shows the impact of a stimulus of fixed amplitude at time 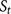 (x axis) using 20 different noise input seeds for the noise vector after the stimulation (vertical axis). See the time series in Fig. 6 (c) for the position of spikes and slow waves. Variations in the successful stimulation timing when using different noise vectors can be seen. For example, using 1 as the noise seed only around a third (32.3%) of the stimulations were successful, whereas when using 18 as the noise seed 95% of stimuli of the same amplitude successfully terminated the SW within 3 seconds.

In order to account for this variability, we use the notion of the stimulation success rate, which is essentially the return probability of the stimulus target position. We calculate the success rate for a stimulus by taking the percentage of successful simulation trials over the total number of simulation trials (20 in our case). For example, the strip at the top of Fig. 6 (c) shows the success rate at different stimulation time points derived from the data in Fig. 6 (b). Essentially, the strip in Fig. 6 (c) is an average of Fig. 6 (b) over the noise trials. When this success rate is mapped onto the time series, some regularity can be observed. It is clear that the top of the wave and the downward part of the wave are typically the most successful periods during the SW cycle (using the same fixed setting of stimulus amplitude and direction as in Fig. 5 (a)). This is to be expected given the results in the deterministic system (see Fig. 5 (a)), as the basin of attraction is, to some extent, preserved under the noise-input (as reported in the second Results section).

So far we have used the same stimulus amplitude in the analysis. When additionally scanning the stimulus amplitude at each stimulation time point, we derive a success rate map for our simulated seizure (see Fig. 6 (d)). The ‘strip’ in Fig. 6 (c) is an extract of the ‘map’ in Fig. 6 (d), indicated by the grey box. The map is effectively a likelihood of success of a stimulus for a specific stimulus amplitude and timing in the simulated seizure. Again, the pattern of high success rate stimulation parameters agree to some extent with the deterministically derived pattern for one cycle of SWD (compare with Fig. 5 (b)).

### Cycle to cycle variation

Despite the agreement of the deterministic stimulation pattern and the noise-driven success rate pattern, a between cycle variability is observed in the noise-driven case in Fig. 6 (c) and (d). For example the pattern for the first full cycle of SWD after 0 s in Fig. 6 (d) is quantitatively different from the first full SW cycle just after 2 s. Although some qualitative similarity can be observed between the cycle patterns, which agrees with the deterministic prediction (Fig. 5 (b)), the variability is strong enough to significantly alter success rates even during the apparently optimal phase around the top of the wave (e.g see the lack of success on top of the wave between 2.1 and 2.4 seconds, in Fig. 6 (c)).

As the success rate is derived from averages over repeated simulations with different noise vectors, these cycle to cycle variations cannot be attributed to the noise effect alone. In order to elucidate the source of the cycle to cycle variations we plot the time series as a trajectory in state space. We shall also map the success rate onto the trajectory with the same colour map as before. Instead of the model-specific state space in Figs. 3 and 5 we now plot the state space reconstructed by time delay embedding because this can also be obtained from an EEG recording and thus allows direct comparison (see Model section 6 for details of attractor reconstruction in state space).

**Figure 7:**
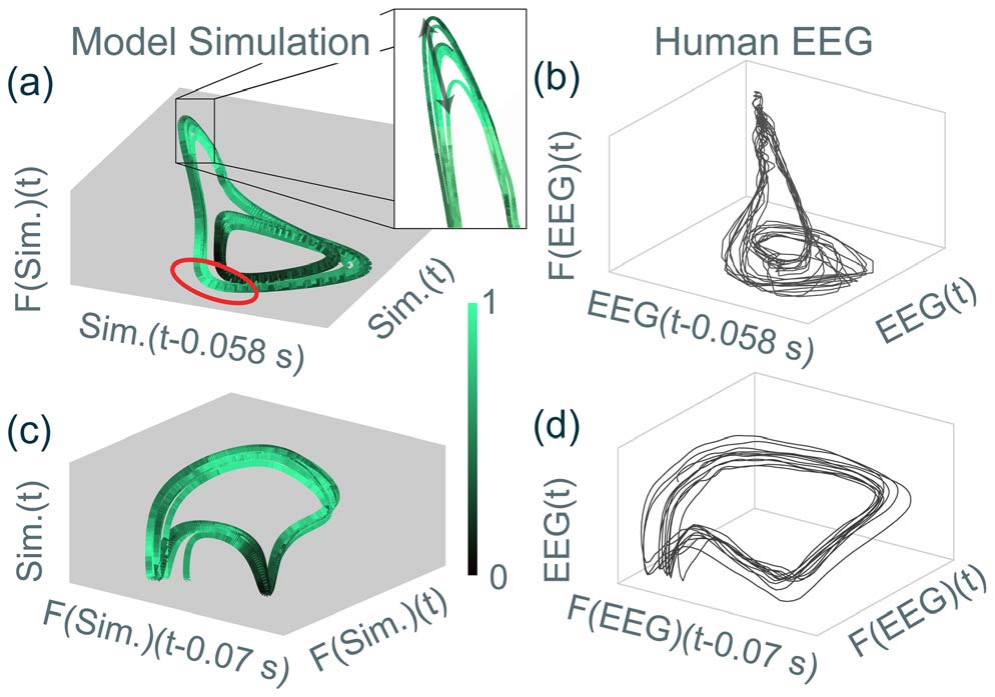
Delay embedding reconstruction of the SWD attractor. The simulated seizure (left) and the clinical seizure (right) are reconstructed. The same reconstruction parameters (delay time and filter frequency cut-off) have been used for both simulated EEGs (Sim.) and clinical EEGs (EEG). F(…) indicates low-pass filtering of the simulated EEG, as explained in the Methods section. Time delays used are indicated in seconds on the axis label. **(a, b)** Reconstructed attractor, in this case corresponding to the 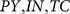 phase space view (c.f. Fig. 5 (a) rotated). **(c, d)** Reconstructed attractor corresponding to the 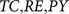 phase space view (c.f. Fig. 5 (c)). 3D Matlab.fig files are available for all subfigures in S5 File, S6 File, S7 File, & S8 File.

Fig. 7 (a) and (c) are reconstructed state space projections using both the unfiltered and filtered model output. Fig. 7 (a) and (b) are comparable to the 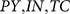 state space, and Fig. 7 (c) and (d) are comparable to the 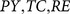 state space. (See Methods for details of the reconstructed phase space.) In this state space view it becomes clear that the exact state space position of the SW trajectory varies from one cycle to the next. This is in contrast to the deterministic case where all cycles follow an identical path. The reason for this variation is that the model (particularly with its slow thalamic compartment) acts as a moderate low-pass filter for the noise.

In consequence, although the change in position is not very strong, the (average) result of stimulation may change considerably. For example, in the zoom-in panel of Fig. 7 (a), the variation of the position along the direction indicated by the black arrow is enough to modify the outcome from mostly successful to mostly unsuccessful. Nevertheless it can be seen that the predominantly successful (circled in red) and unsuccessful regions cluster in state space and optimal stimulation regions can be identified. This indicates that the altered position of the cycle changes the relative position of the basin of attraction (or region of high return probability), and consequently leads to altered mean success rates for the same stimulus from the same SW phase.

For comparison, we plotted a reconstructed state space from a clinical EEG recording during a SW seizure in Figs. 7(b) and (d). There is good qualitative agreement between the simulated and clinical SW cycle forms, also in terms of the cycle to cycle variation. In order to determine the success rate in the clinical scenario, we suggest that a patient-specific derivation should be performed (as discussed later). The model derived high success rate regions are not necessarily directly applicable to the clinical case, as (i) the stimulus direction is arbitrarily chosen in the model and (ii) the model parameters are not patient-specific, hence the basin of attraction cannot be expected to be identical in the model as in the patient.

Nevertheless, our presented results are still highly relevant, as we proposed mechanistic reasons for the failure of current stimulation protocols in SW seizures. For example, we could show that when using arbitrary stimulation timings and amplitude, an average success rate of about 55% can be obtained (average success rate of the whole map in Fig. 6 (d)). This would agree with the success rate of 57% reported in [8] (where stimulation was delivered at arbitrary time points during the cycle with potentially varying amplitude). Even with an optimised stimulation amplitude, only a maximal success rate of 65% was obtained. Only by taking into account all the mechanisms we proposed in this study, optimal stimulation regions in state space can be derived (red circle in Fig. 7). In Discussion, we propose how this mechanistic insight should be used to devise better stimulation protocols.

## Discussion

In this study we have treated generalised spike-wave seizures from a dynamical systems point of view. Abrupt changes in dynamics associated with disease states have been suggested as evidence that such a perspective might complement more traditional views of disease pathophysiology [27]. Anecdotal evidence that absence seizures can be terminated by a brief acoustic stimulus (i.e. by an auditory or arousal mechanism) was considered as a hint that SW seizures might present an example of a bistability between a background and a disease state [24]. Such a concept has more recently been connected to other abnormal states as well [28].

Whilst the proposal of single-pulse abatement of SW seizures [24] was implemented in a computational model of the thalamo-cortical loop [22], the model in that study did not display the characteristic spike-wave waveform and the full geometry of the basin of attraction was not studied. Our work shows that when a realistic waveform is considered, even in the simplified version of the thalamo-cortical loop the response to stimulation is much more complex.

Our model eq. (4) produces a 4D basin of attraction of the background state, which we visualised as a time-varying 3D projection. The basin of attraction is well-defined in the case of deterministic simulations where noise input is ignored. Nevertheless, we demonstrated that the basin is a complicated structure in our model that leads to a non-trivial phase- and amplitude-dependency of the stimulus (Fig. 5 and S1 Movie). Specifically, in different phases of the SW oscillation, a stimulus with the same strength (amplitude) might either abort the SW sequence, leave it unaffected, or even prolong it. Previous experimental [29] and clinical [30] studies have suggested phase dependency as being crucial for stimulation success. However, unlike in cases where the pathological dynamics is a simple sinusoidal oscillation (see e.g. the phase dependence of stimulating an essential tremor rhythm [31]), the SW morphology is associated with a basin boundary which manifests as highly complex geometrical object. Nevertheless, a clear pattern of successful stimulation parameters can be estimated from the scan of phase and amplitude of the stimulation (Fig. 5).

A second level of complication comes with the addition of noise input to the model. Noise is unavoidable in the *in vivo* situation. In the bistable model it is used as the driving mechanisms for spontaneous transitions into and out of SW seizures. The presence of noise makes the borders of the basin of attraction fuzzy (Fig. 4). Nevertheless, the noise input in our model leaves the deterministic structures intact, as the dynamics of the SW seizures was shown to be dominated by deterministic behaviour [26]. Hence, the predominantly deterministic nature of SW seizures are likely to preserve the core of the basin, i.e. a robust region in phase space into which the dynamics can be directed to abort the seizure.

However, there is a third level of complication. Due to the low-pass filtering properties of the slow time scale on the noise input, the position of the SW cycle relative to the basin of attraction may vary substantially from cycle to cycle. With the complex geometry of the basin, this leads to a significant alteration of the degree of success one can expect from repeated application of the same stimulus in the same SW phase. As the exact geometry of the basin will be unknown in the clinical setting, it is unlikely that any fixed set of successful stimulation parameters can be predicted even with a detailed model of SW.

**Figure 8:**
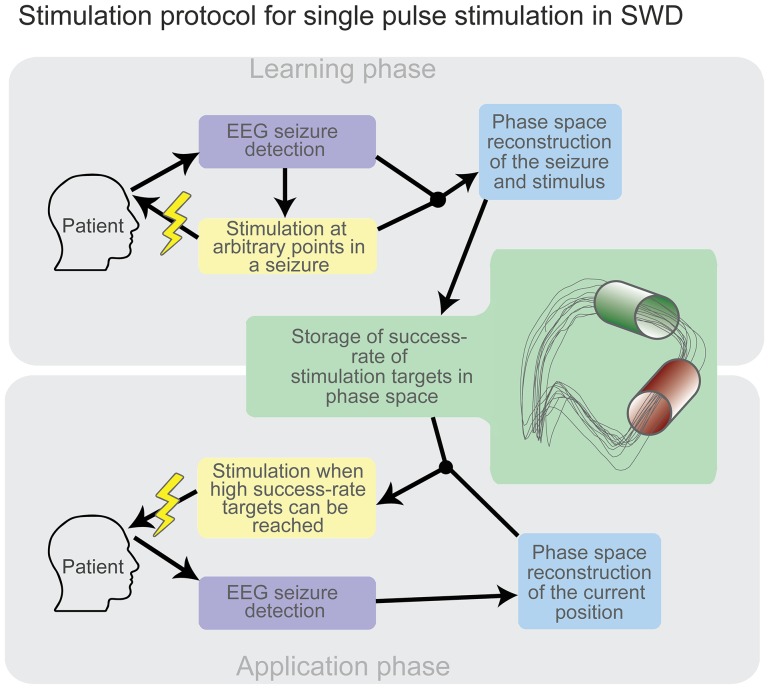
Schematic of a suggested single pulse stimulation protocol. In the learning phase (top) success rate of state space targets are stored based on arbitrary stimulations during seizures. Once the state space is charted, the application phase (bottom) can use the information of the success rate of state space targets to deliver high success rate stimuli to abate SWD seizures.

We therefore suggest a practical solution to the problem of determining stimulation parameter candidates using a state space approach. A low dimensional state space can be reconstructed from a single clinical recording using delay embedding. In the learning phase, the result of a stimulation at a given point in state space is stored. Stimuli of different amplitudes can be used to reach better coverage of the state space. When a state space volume has reached a certain density in terms of stored points, a return probability of this volume can be calculated (see Fig. 8). Repeated stimulation should then lead to the appearance of volumes associated with high return probability in a given patient. Note that in the case of no success, stimulation can be repeated within the same seizure. Also the frequency of typical absences and the fact that they can often be precipitated by hyperventilation should allow for sufficient trials in the learning phase to estimate a volume in phase space that is the best target for single-pulse stimulation. The consistency of SW seizures within patients as compared to the variability between patients [19] leads us to expect that each patient will have individual optimal state space volumes. Therefore adaptable algorithms based on the real-time determination of position in a reconstructed state space are the optimal strategy according to our computational study.

Such a stimulation protocol could be implemented in an “automatic self-stimulation” device, as suggested by [8]. In such a device, surface electrodes can be used for the detection of SWD in real time and stimulation can be delivered, for example, by an auditory stimulus. The closed loop stimulation device can be entirely non-invasive in design and implementation. For validation of the stimulation protocol on SWD in animal models the WAG/Rij or GAERS rats could be used experimentally [6], [32]. Due to the non-invasive nature this approach could also be tested during clinical monitoring or during sleep in a non-clinical setting. For other types of seizures, invasive designs (as successfully applied in animal models [5], [6], [33]) might be adopted.

In the current work we only investigated the effect of single pulse stimulation and assumed that these pulses have the effect of directly influencing the state variables (i.e. variables representing the EEG voltage) and preserving the bifurcation structure. This is conceptually different to bifurcation control (e.g. [34], [35]), where it is assumed that a particular system parameter can be controlled/tuned from outside. In the latter scenario, the SW attractor can be destroyed altogether by shifting a parameter out of the bistable region in parameter space. Such an approach requires more physiological details to be considered in modelling studies and might therefore best be done in a detailed biophysical model of SW, e.g. [36].

One potential limitation of the model in its current presentation is that any spatial interactions are lumped together (as in previous cases, e.g. [11]). On the one hand, it has been argued for generalised absence seizures that spatially extended brain processes may be responsible mechanisms and consequently a reduction in the spatial dimension [37] can be made. On the other hand, it was demonstrated that spatial heterogeneities could be important for seizure genesis and maintenance [13], [38], [39]. Future work should include such heterogeneities, ideally using patient-derived connectivity data as suggested in [40], [41], to additionally investigate the optimal stimulus position in space [42].

In summary, our study predicts that SWD seizures can be abated through the application of single pulse stimulation. Successful stimulation requires that the optimal pulse targets a specific region in state space: the basin of attraction of the background behaviour. Due to the complexity of the basin and the relative position of the SW trajectory to it, the optimal stimulation timing and amplitude is predicted to be complex and time-dependent. We suggest that the real-time use of a reconstructed state space can aid a learning/optimisation algorithm in a patient-specific manner. Such an adaptive algorithm could then potentially be used to non-invasively suppress generalised SW activity, particularly in paediatric patients.

## Materials and Methods

### Physiological basis of the model

The bilateral generalised nature of many SWD seizures has led many investigators to hypothesise about an underlying pacemaker to synchronise such large cortical areas. Indeed, experimental and clinical evidence suggests a key role for thalamic involvement (see eg. [43] and references therein) in widespread SWD.

**Figure 9:**
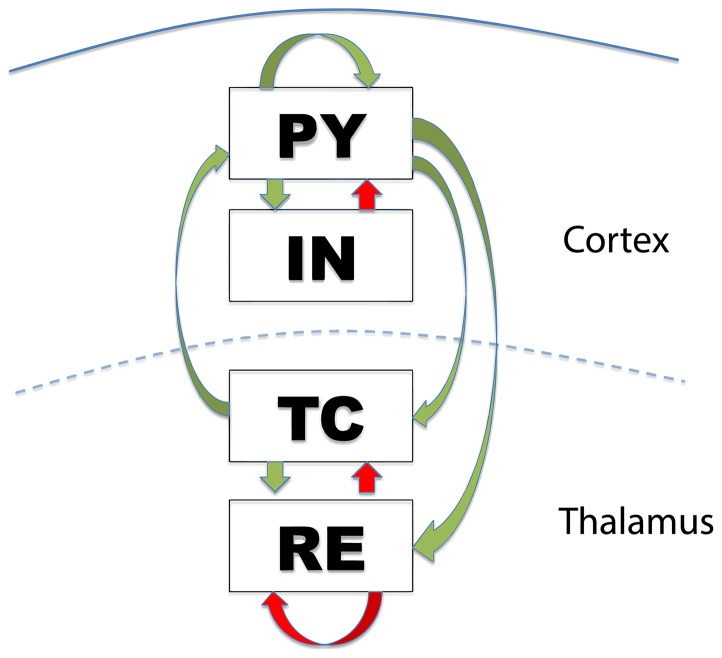
Connectivity scheme of the model. Excitatory (inhibitory) connections indicated in green (red). 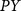 is the cortical pyramidal neural population, 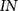 is the cortical inhibitory neural population, 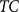 is the thalamocortical neural population, and 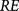 is the thalamic reticular nucleus neural population.

To model thalamocortical interactions we follow previous modelling approaches based on the physiological connectivity of this system (see Fig. 9 below, and compare to [22] and [12]). Specifically, the neural mass approach by [22] forms a neural population version of the detailed biophysical model advanced by [36]. On the macroscopic level, the pyramidal cell population (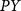) variable is self-excitatory [44] and excites the inhibitory interneuron population (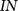) [22]. In addition, 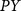 excites thalamocortical cells in the thalamus (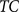), and cells in the reticular nucleus of the thalamus (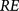) [22], [45]. Inhibitory interneurons inhibit local cortical 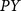 cells only [22]. Direct thalamic output to the cortex comes exclusively from excitatory 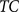 connections to 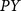 populations [12]. Intrathalamic connectivity is incorporated into the model as follows: 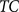 cells have excitatory projections to 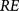, which in turn inhibits the 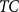 population along with self-inhibition of 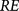. This connectivity scheme is consistent with experimental results reviewed in [16] and summarised in their Fig. 1.

Finally, we incorporate a slow timescale into the thalamic compartment, as it has been demonstrated in a minimal model of SWD that at least a slow driver is required in addition to the 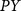 and 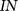 units [15], which we assume to be the cortical populations that generate the SWD seizure EEG [46]. Furthermore, there is experimental evidence for abnormal slow processes (variations in a tonic inhibitory current), which may be a common mechanism in typical absence seizures [47]. This is also supported by theoretical studies that find slow timescales crucial for the generation of realistic SWD. These studies either incorporate the slow timescale directly by modelling the slower reaction of thalamic populations [39], [48] or by incorporating explicit delays [12]. [48] compares the two approaches and finds similar bifurcation structures leading to the onset of SWD. As the exact dynamic mechanisms underlying the emergence of the slow timescale is still unclear, we assume that the thalamic compartment operates on a slower timescale. This specifically has the advantage that the model could in future be analysed in terms of slow-fast subsystems [15].

### Deterministic Model

We implement the model using a neural population version of the Amari neural field equations [44], following [14], [39]. The explicit connectivity scheme and a formal description of the model including the equations are given below.

For simulations of the deterministic system the ode45 MATLAB solver was used with Eqn. 1. 
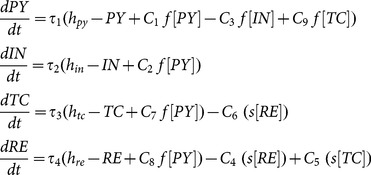
(1)


where 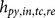 are input parameters, 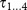 are time scale parameters and 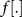 and 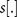 are the activation functions: 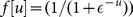
(2)

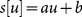
(3)


with 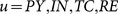. The parameter 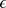 determines the sigmoid steepness.

These equations implement the connection schematic as shown in Fig. 9. The model EEG is taken as the mean of the two cortical populations. All model parameters used to produce the figures in this manuscript are listed in Tab. 1. In this study we place the model in a bistable state. Parameter scans of the input parameters to the TC and RE variables indicate that the bistability occupies a fairly large region in the SWD parameter space (S1 Fig.) and can therefore be considered robust.

**Table 1:** Parameter values used to produce the figures in this manuscript.

Parameter	Interpretation	Fig. 2, 4b, 6, 7	Fig. 3, 4a, 5
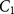	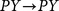 connectivity strength	1.8	1.8
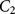	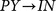 connectivity strength	4	4
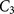	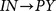 connectivity strength	1.5	1.5
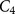	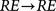 connectivity strength	0.2	0.2
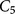	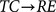 connectivity strength	10.5	10.5
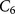	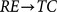 connectivity strength	0.6	0.6
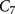	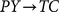 connectivity strength	3	3
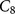	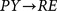 connectivity strength	3	3
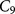	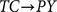 connectivity strength	1	1
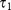	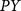 timescale	26	26
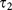	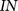 timescale	32.5	32.5
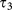	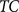 timescale	2.6	2.6
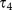	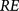 timescale	2.6	2.6
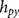	Input 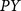	−0.35	−0.35
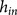	Input 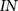	−3.4	−3.4
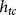	Input 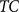	−2.05	−2.0
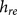	Input 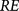	−5	−5
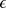	Sigmoid steepness	250000	250000
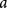	Linear intersection steepness	2.8	2.8
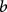	Linear intersection offset	0.5	0.5
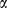	Standard deviation of noise	0.022	0

For simplicity, a linear activation term 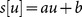 is used instead of the sigmoid (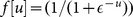) is used in the thalamic subsystem. This approximation is justified because the thalamic compartment is mainly operating in the linear range of the sigmoid for the SWD. It simplifies the analysis of the model and the adjustment of the required bistability between background activity and SW dynamics. S2 Fig. shows the qualitative agreement between the two versions of the model (with sigmoidal and linear activation functions for the thalamic compartment) including the existence of bistable SWD upon perturbation at 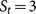.

### Stochastic Simulations

We also use a stochastic equivalent of the model in Eqn. 1 to simulate noise driven seizure transitions. The noise term was added to the TC population following previous modelling literature of the thalamo-cortical loop [11], [12], [48], and represents non-specific ascending noise input from the brain stem. The noise driven model is written as a stochastic differential equation:
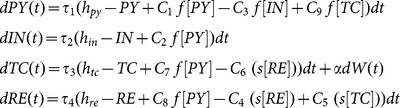
(4)


Simulations were performed with Eqn. 4 using the Euler-Maruyama solver and a step size (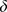) of 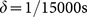. Simulations using smaller step sizes yielded qualitatively similar results. Autonomous seizure transitions occur in the model as a result of added noise to the TC variable in line with [48]. The noise-term 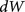 follows a normal distribution with zero mean and standard deviation 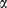. The higher the noise amplitude 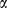 is, the more frequently shorter seizures occur. We adjusted 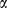 such that seizures of about 5-10 seconds occurred every few minutes, in accordance with clinical findings. All solutions were checked for stability using alternative solvers and were found to be qualitatively robust. Parameter values used can be found in Table 1.

### Reconstruction of the basin of attraction

To numerically determine the deterministic basin of attraction of the background state, we systematically scan the initial conditions of the simulations in the four dimensional state space. The basin is obtained with our motivation to study perturbations of the seizure state. Hence, during a simulation of the SW we fix one of the variables at a time point (indicated by the red star in Fig. 3 (a)) and scan the state space of the three remaining variables. We record the state space initial condition points from which the trajectory reaches the background state within 3 s. We define a return to the background state as a stimulus which ensures the model output does not exceed a threshold of 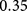 for more than 3 seconds post stimulus. The threshold is used as a heuristic to detect high amplitude SW oscillations in our model. This measurement is aimed to reflect what could be done experimentally (i.e. inducing sub-threshold, low amplitude activity). By scanning many time points on a SW cycle for a fixed variable, we obtain multiple 3D slices of the 4D basin. The scan resolution (in state space, as well as in time) chosen was determined by the available computational power.

Fig. 3 and Fig. 5 (c,d) used 3D slices in the 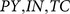 dimensions and the scan points belonging to the basin are marked with coloured dots. S1 Movie shows the basin of attraction, with the fourth dimension (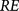) mapped to the time domain. In the video, we have also included the critical manifold [49] of the cortical subsystem as a blue grid for orientation. For more details regarding critical manifolds in SW, see [15].

Under noise-input, the basin of attraction is not well-defined. Trajectories starting near the (former) basin boundary can either reach the steady state or the SW state depending and the noise input. We therefore determine the likelihood of trajectories to reach the background state by repeating the simulation for each scanned state space point with different noise vectors. Similar to the deterministic case we deem an initial condition as belonging to the basin, if the trajectory approaches the noisy background state within 3 s. From averages over 20 noise trials for each state space point, we derive a likelihood of the scanned state space points to belong to the basin of attraction (i.e. a probability to return to the background state).

This definition of return probability of state space points is compatible with the deterministic basin of attraction with its well-defined separatrix. There, all points within the basin have a return probability of one, all points outside have a return probability of zero.

### Simulation of single pulse stimulation

We are interested in using our model to better understand the results of stimulation for the abatement of spike-wave seizures. Single pulse stimulation at time 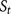 was performed by simulating the model (stochastic or deterministic) from 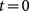 to 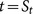, then changing the variables to be stimulated by the desired stimulus amplitude 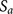 and then continuing the simulation from 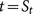 to 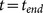. (I.e. an initial condition reset.)

In the experimental setting the direction of the stimuli is not necessarily controllable (e.g. a TMS pulse), if it were the problem of stimulation is trivial and the background fixed point could always be targeted. We therefore keep the stimulation direction constant, with equal input to the 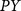 and 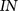 variables, thus simulating an unspecific stimulation to the cortex.

In order to gauge whether a stimulus in the model was successful, we measure the distance of the trajectory to the background fixed point 3 s after the stimulation and define that the stimulus is successful if the trajectory returns to the vicinity of the fixed point.

### Attractor reconstruction

In order to compare clinical data with model simulation, we not only show comparison of time series, but also comparison of attractors in state space. To recover the attractor from clinical time series in a state space comparable to the model state space, we assume that the dynamics are dominated by a deterministic behaviour (as in, e.g. [12]). In this case delay embedding can be used [50]. Furthermore, to recover the slow variables in a fast-slow system, [51] suggests using a low pass filter in conjunction with the above. Since our model contains fast (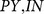) and slow (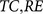) variables we use the same technique of delay embedding and low pass filtering to reconstruct a comparable attractor (to our model) from clinical and simulated time series (compare for example the seizure attractor in Fig. 7a,c with the clinically recorded reconstruction in Fig. 7b,d).

To reconstruct the attractor from the time series there are two key parameters. Firstly the low pass filter cutoff must be chosen such that the fast frequencies are removed. Secondly, the delay must be chosen to provide good visibility of the structure. In this study a low-pass filter is used with a cutoff at 6 Hz and time delays are chosen on a per figure basis (all approximately 0.06 s).

### MATLAB code

Matlab code and parameters for the model will be made available online at modelDB.

## Supporting Information

S1 Fig
**Parameter scans of input parameters to the thalamic subsystem in the deterministic system.** Parameter scans showing bistability between background fixed point and SWD limit cycle scanning 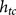 (a), 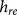 (b). Bistable regions are highlighted in grey.(TIF)

S2 Fig **Comparing linear and sigmoidal activation functions in the thalamic subsystem.** Model dynamics are qualitatively similar using either the linear activation function (a) or the nonlinear sigmoid function (b) in the thalamic subsystem. The system in both cases is bistable and a perturbation at t = 3 s induces a transition from the fixed point to the SWD attractor.
(TIF)

S1 File
**3D Matlab figure for** **Fig. 3 (a)** **.** 3D Matlab figure file showing the 3D slice of the basin of attraction of the background fixed point in the 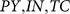 state space. Black solid line indicates the SWD attractor. Additional state space structures can be made visible in the figure editor.(ZIP)

S2 File
**3D Matlab figure for** **Fig. 3 (b)** **.** 3D Matlab figure file showing the 3D slice of the basin of attraction of the background fixed point in the 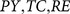 state space. Black solid line indicates the SWD attractor. Additional state space structures can be made visible in the figure editor.(ZIP)

S3 File
**3D Matlab figure for** **Fig. 5 (c)** **.** 3D Matlab figure file showing the 3D slice of the basin of attraction of the background fixed point in the 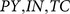 state space. The successful stimulation at this point is shown in cyan. Black solid line indicates the SWD attractor. Additional state space structures can be made visible in the figure editor.(ZIP)

S4 File
**3D Matlab figure for** **Fig. 5 (d)** **.** 3D Matlab figure file showing the 3D slice of the basin of attraction of the background fixed point in the 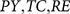 state space. The unsuccessful stimulation at this point is shown in red. Black solid line indicates the SWD attractor. Additional state space structures can be made visible in the figure editor.(ZIP)

S5 File
**3D Matlab figure for** **Fig. 7 (a)** **.** 3D Matlab figure file showing the 3D projection of the reconstructed simulated SWD trajectory in a 3D delay embedding. This embedding is comparable to the 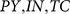 state space. The colour map at any point indicates the success rate of a stimulus at this point (of amplitude -0.0825).(ZIP)

S6 File **3D Matlab figure for** **Fig. 7 (b)** **.** 3D Matlab figure file showing the 3D projection of the reconstructed clinical SWD trajectory in the same 3D delay embedding as S5 File.
(ZIP)

S7 File
**3D Matlab figure for** **Fig. 7 (c)** **.** 3D Matlab figure file showing the 3D projection of the reconstructed simulated SWD trajectory in a 3D delay embedding. This embedding is comparable to the 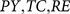 state space. The colour map at any point indicates the success rate of a stimulus at this point (of amplitude -0.0825).(ZIP)

S8 File **3D Matlab figure for** **Fig. 7 (d)** **.** 3D Matlab figure file showing the 3D projection of the reconstructed clinical SWD trajectory in the same 3D delay embedding as S7 File.
(ZIP)

S1 Movie
**The four dimensional basin of attraction shown in 3D slices over time.** The 3D slices are in 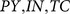 state space, changing over time, essentially mapping 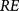 onto the time domain. The 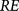 slice points were chosen to be the 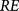 values of the SWD attractor. Additionally, the critical manifold [49] is shown as a blue grid for orientation. For more details regarding critical manifolds in SW, see [15].(MOV)
