# Supplementary figures and images for: A Computational Study of Stimulus Driven Epileptic Seizure Abatement
Source: PLoS One. 2014 Dec 22;9(12):e114316. doi: 10.1371/journal.pone.0114316 (PMC4273970; doi:10.1371/journal.pone.0114316)

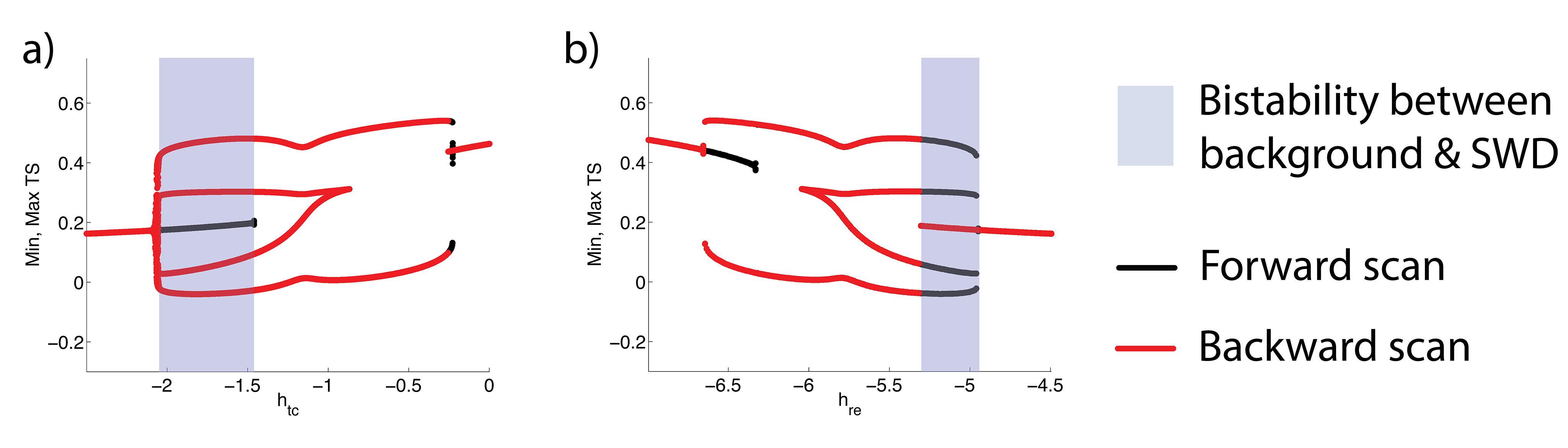

Supplement: S1 Fig — Parameter scans of input parameters to the thalamic subsystem in the deterministic system. Parameter scans showing bistability between background fixed point and SWD limit cycle scanning (a), (b). Bistable regions are highlighted in grey. (TIF) [file pone.0114316.s001.tif]

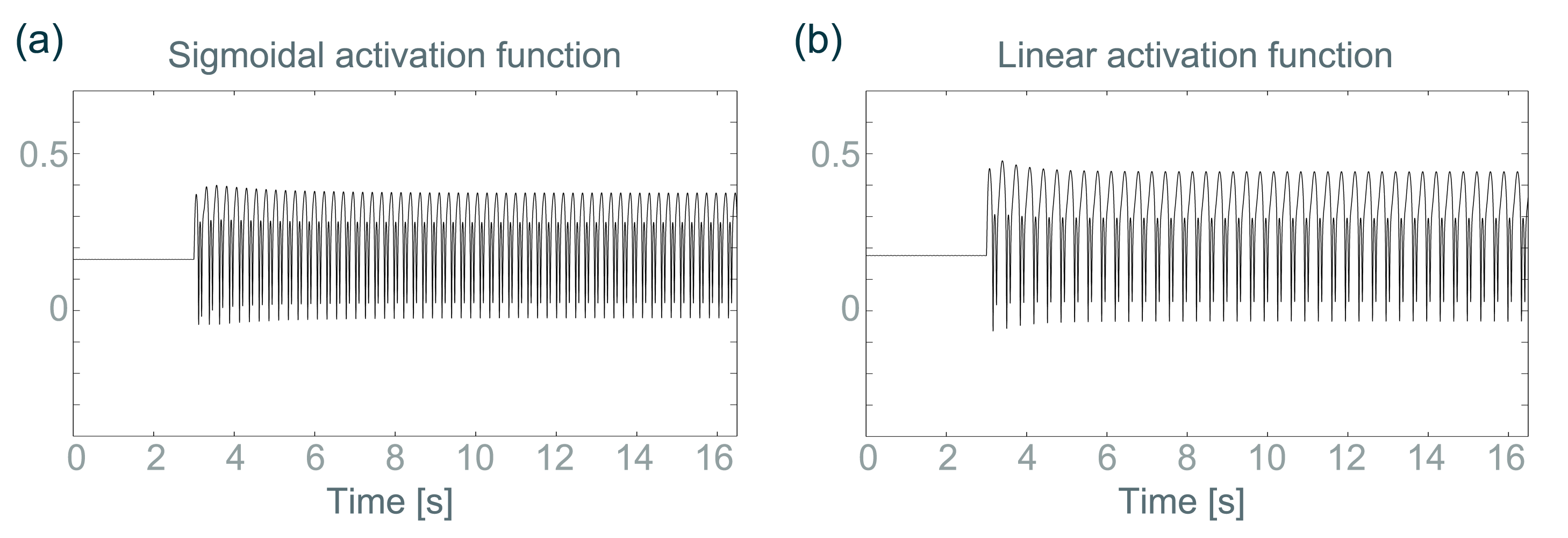

Supplement: S2 Fig — Comparing linear and sigmoidal activation functions in the thalamic subsystem. Model dynamics are qualitatively similar using either the linear activation function (a) or the nonlinear sigmoid function (b) in the thalamic subsystem. The system in both cases is bistable and a perturbation at t = 3 s induces a transition from the fixed point to the SWD attractor. (TIF) [file pone.0114316.s002.tif]
